# The long‐term effects of childhood circumstances on older individuals: A systematic review

**DOI:** 10.1002/agm2.12299

**Published:** 2024-04-12

**Authors:** Mst. Rina Parvin, Fateha Tuj Johra, Fazila Akter, Md. Wahiduzzaman, Khadiza Akter, Mousumi Das, Sujit Mondal, Mitun Debnath, Mohammad Ullah, Moustaq Karim Khan Rony

**Affiliations:** ^1^ Major at Bangladesh Army (AFNS Officer), Combined Military Hospital Dhaka Dhaka Bangladesh; ^2^ School of Medical Sciences Shahjalal University of Science and Technology Sylhet Bangladesh; ^3^ Masters in Disaster Management University of Dhaka Dhaka Bangladesh; ^4^ Dhaka Nursing College, affiliated with the University of Dhaka Dhaka Bangladesh; ^5^ Master of Public Health Daffodil International University Dhaka Bangladesh; ^6^ Master of Public Health Leading University Sylhet Bangladesh; ^7^ Master of Science in Nursing National Institute of Advanced Nursing Education and Research Mugda Dhaka Bangladesh; ^8^ Master of Public Health National Institute of Preventive and Social Medicine Dhaka Bangladesh; ^9^ College of Nursing International University of Business Agriculture and Technology (IUBAT) Dhaka Bangladesh; ^10^ Master of Public Health Bangladesh Open University Dhaka Bangladesh

**Keywords:** childhood experiences, early‐life, health, older individuals

## Abstract

Childhood experiences are known to shape individuals' development and can influence various aspects of life later on. Understanding the long‐term effects is crucial for informing interventions and policies aimed at promoting healthy aging. This review aimed to explore the long‐term effects of childhood experiences on older individuals. This systematic review comprised three distinct phases. Firstly, a systematic review was conducted, exploring databases such as Google Scholar, PubMed, EMBASE, PsycINFO, and the Web of Science. Out of the 2116 studies initially identified, 24 studies were selected based on the inclusion criteria. Secondly, these inclusion criteria were applied to ensure that the chosen studies specifically delved into the connection between childhood experiences and outcomes in older individuals. Finally, data extraction and synthesis techniques were employed to analyze findings, facilitating the drawing of conclusions concerning the enduring impacts of childhood experiences on the well‐being of older individuals. The review's findings revealed how negative experiences in childhood continue to affect older individuals in various ways. These early‐life events have far‐reaching consequences, profoundly impacting their physical health, making them more susceptible to chronic diseases and weakening their immune system. Additionally, they affect mental health, leading to conditions like depression, anxiety, and substance abuse. Cognitive function is also affected, resulting in memory problems and cognitive decline. Furthermore, these experiences impact social relationships, affecting trust, emotional control, and social isolation in later life. This review highlighted the enduring influence of childhood circumstances on the health and well‐being of older individuals. Policymakers and health care practitioners should consider these findings when developing strategies to support healthy aging and mitigate the long‐term effects of adverse childhood experiences.

## Graphical abstract

Childhood experiences profoundly shape the health, mental well‐being, cognitive function, and social relationships of older individuals. From malnutrition and environmental pollutants to trauma and socioeconomic factors, early life significantly influences later‐life outcomes, emphasizing the importance of comprehensive support and interventions for the older people.

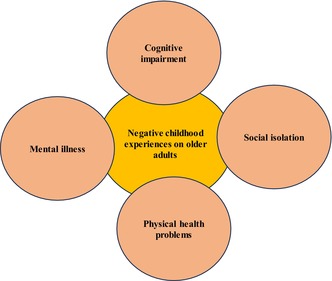

## 1 BACKGROUND

Every facet of our childhood significantly contributes to our future, impacting our older individuals' health and our behavior, emotional well‐being, and thought processes. The circumstances of our early years leave lasting impressions, influencing our health trajectory in older individuals. The quality of parenting and a child's attachment style notably impact both physical and mental health in the long term.^1^, ^2^ Establishing a secure attachment, characterized by a caregiver providing consistent support, responsiveness, and emotional availability, fosters emotional resilience. This secure attachment, in turn, improves stress management in older individuals and diminishes the likelihood of mental health disorders and stress‐induced physical conditions.^3^ Moreover, socioeconomic status (SES) during childhood significantly shapes health outcomes in later life. Children from lower SES backgrounds often face greater adversity, including limited access to health care, nutrition, and educational opportunities, which can exacerbate health disparities.^4^

Schools also wield considerable influence over the health of older individuals. They serve a vital role in socialization that extends beyond imparting education. Early childhood illnesses indirectly impact the well‐being of older individuals by disrupting their school attendance and performance, potentially limiting their future educational and employment opportunities, as well as their overall health status.^5^, ^6^ Moreover, specific childhood illnesses can have enduring effects on the health of older individuals.^7^ For example, children with chronic conditions like asthma may encounter related health issues as they advance in age. Consequently, positive experiences contribute to good mental health, while negative experiences, such as bullying, can lead to persistent mental health issues.^8^ Within this context, the influence of peers during childhood and adolescence plays a pivotal role in shaping behaviors that influence the health of older individuals, encompassing their dietary choices, physical activity, and the consumption of substances such as alcohol and drugs.^9^

Inactive behaviors during childhood, such as prolonged screen time or a lack of outdoor play, can set a pattern for a comparable lifestyle in older individuals, increasing the chances of obesity, cardiovascular disease, and other health issues.^10^, ^11^ In contrast, children who consistently participate in physical activities are more likely to carry on with this healthy practice into their later years.^12^ Furthermore, broader community and cultural factors have a notable impact on long‐term quality of life.^13^ For example, cultural perspectives on food and physical activity can mold habits that last a lifetime.^14^ Safety and unity within a community also exert both immediate and enduring influences on mental well‐being.^15^ However, understanding the long‐term effects of childhood experiences on older people's health is a critical area of public health research. It integrates the fields of epidemiology, psychology, sociology, and medicine to give a whole picture of how one's upbringing can have a lasting impact on one's health throughout their entire life. As a critical developmental phase, childhood sets the foundation for health, well‐being, and productivity in older individuals.

The rationale for conducting this review is threefold. Firstly, it synthesizes findings from various fields of study, fostering an interdisciplinary understanding of how early‐life experiences shape the health of older individuals. This consolidates the existing knowledge base, shedding light on potential interactions among different childhood factors and their cumulative impact on older people's health. Secondly, it identifies areas that require further research, uncovers gaps in our current understanding, and prompts more nuanced future investigations. Moreover, it offers valuable insights to inform public health policies, health care provisions, and early intervention strategies. By emphasizing the long‐term effects of childhood experiences on health, it highlights the need for preventive measures and early intervention efforts. Therefore, this review aims to investigate the enduring consequences of childhood experiences on older individuals.

## 2 METHODS

### 2.1 Search strategy

**FIGURE 1:**
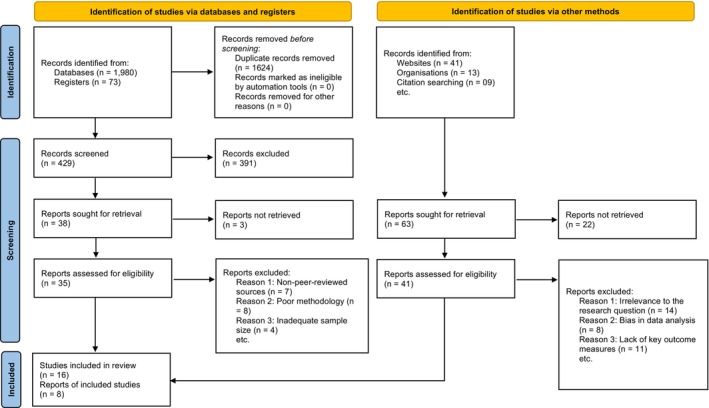
PRISMA flow diagram.^7^

Web of Science databases, such as Google Scholar, PubMed, EMBASE, PsycINFO, and Web of Science, were utilized to conduct a comprehensive literature search (Figure 1). The search strategy was designed to encompass all research studies that examined the impact of childhood experiences on older individuals' health outcomes over an extended period. Key search terms in our strategy encompassed ‘childhood circumstances,’ ‘childhood experiences,’ ‘long‐term effects,’ ‘elderly individuals,’ and ‘older adults’ health.

To ensure a comprehensive retrieval of relevant literature, our search string incorporated both Medical Subject Headings (MeSH) terms, which are standardized vocabulary used for indexing articles in databases, and keywords, which are specific words or phrases relevant to the research topic.^16^ This approach enhances the search strategy's effectiveness and facilitates the identification of pertinent studies across various databases. For instance, our search string on PubMed consisted of (“childhood circumstances” [MeSH Terms] OR “childhood experiences” OR “childhood adversity” [All Fields]) AND (“long‐term effects” [All Fields] OR “life‐course” [All Fields]) AND (“older adults” [All Fields] OR “elderly health” [All Fields] OR “health outcomes” [All Fields]). Additionally, the reference lists of all included articles were manually searched to identify any potentially relevant studies that may have been missed during the initial database search.

### 2.2 Study selection criteria

After conducting the database search, duplicate records were removed using reference management software. Subsequently, the researchers independently evaluated all titles and abstracts based on predetermined inclusion and exclusion criteria. Studies meeting the following criteria were included: (1) being primary research articles published in English between 2017 and 2023, ensuring the incorporation of recent research, and providing a comprehensive overview of current trends and understandings in the field; (2) focusing on childhood experiences, enabling a direct examination of their impact on older individuals' health status, and offering insights into how early‐life events shape health outcomes later in life; and (3) examining their effects on older individuals' health status, ensuring the relevance of the review in exploring the influence of early‐life factors on health outcomes in older age. Non‐empirical studies, such as reviews, editorials, and case studies, as well as those not specifically addressing the impact of childhood experiences on older people's health, were excluded. The review included 24 primary research studies, comprising 16 published as original research studies and 8 published as reports.

### 2.3 Data extraction process

Data were extracted from the selected studies using a standardized data extraction form specifically developed for this review. The form consisted of the following fields: authors, year of publication, study location, study design, sample size, participants' age, description of childhood experiences, older individuals' health outcomes assessed, and key findings. Three researchers independently performed the data extraction from each study, and they cross‐verified their entries to ensure accuracy. In addition, disagreements among researchers during the selection process were resolved through discussions.

### 2.4 Quality appraisal

The process of quality appraisal was carried out meticulously to assess the methodological rigor of each study that met our inclusion criteria. This assessment was conducted independently by three researchers to avoid bias and enhance accuracy. To standardize the quality evaluation process, we utilized the Newcastle‐Ottawa Scale (NOS), a widely recognized tool specifically designed for appraising the quality of non‐randomized studies, including observational studies.^17^ The NOS is based on three broad domains. The first domain pertains to the selection process of study groups. This domain evaluates whether the groups selected for the study are representative of the average population and whether the cases and controls are appropriately matched. The second domain assesses the comparability of study groups, taking into consideration whether the researchers have accounted for other factors that may influence the outcome. These factors could include confounding variables or other variables that the researchers have either controlled or adjusted for in their analysis. The third domain, outcome assessment, examines how the outcomes of interest were measured and whether the duration of follow‐up was sufficiently long to be considered reliable.

The NOS consists of eight items distributed across these three domains (Table 1). Each study was evaluated based on these eight items and assigned a score out of a maximum possible nine points. The points were assigned according to the methodological strength of the study in relation to each item. To classify the quality of the studies, we adhered to a standardized scoring system. Studies scoring seven points or above were classified as high quality, indicating robust design, execution, and analysis. Studies scoring between five and six points were considered of medium quality, signifying some methodological strengths along with noticeable weaknesses or areas of uncertainty. Studies scoring below five were categorized as low quality, indicating significant methodological limitations or a high risk of bias.

**TABLE 1:** Newcastle‐Ottawa Scale (NOS) for quality assessment.

Study ID	Selection of study groups (up to four points)				Comparability of study groups (up to two points)	Outcome assessment (up to three points)			Total score
	Domain 1: Item 1 Representativeness of the exposed cohort (0–1 Point)	Domain 1: Item 2 Selection of the non‐exposed cohort (0–1 Point)	Domain 1: Item 3 Ascertainment of exposure (0–1 Point)	Domain 1: Item 4 Demonstrating that outcome of interest was not present at the start of the study (0–1 Point)	Domain 2: Item 5 Comparability of cohorts on the basis of the design or analysis (0–2 points)	Domain 3: Item 6 Assessment of outcome (0–1 point)	Domain 3: Item 7 Was follow‐up long enough for outcomes to occur (0–1 point)	Domain 3: Item 8 Adequacy of follow‐up of cohorts (0–1 point)	
Study 1	√	√	√		√	√	√		6
Study 2	√	√	√	√	√	√	√	√	8
Study 3	√	√	√	√		√	√	√	7
Study 4	√	√	√	√	√		√	√	7
Study 5	√	√		√	√	√	√	√	7
Study 6	√	√	√	√	√	√	√		7
Study 7	√		√	√	√	√		√	6
Study 8	√	√	√		√	√	√	√	7
Study 9	√		√	√	√	√	√	√	7
Study 10	√	√	√	√	√		√		6
Study 11		√	√	√	√	√	√	√	7
Study 12	√	√	√	√	√	√	√	√	8
Study 13	√	√	√	√		√	√	√	7
Study 14	√	√	√	√	√	√	√	√	8
Study 15	√	√	√	√	√	√	√	√	8
Study 16	√	√	√	√	√	√	√		7
Study 17	√		√	√	√	√	√	√	7
Study 18	√	√	√		√	√	√	√	7
Study 19	√	√	√	√	√	√	√	√	8
Study 20	√		√	√	√	√	√	√	7
Study 21	√	√	√	√	√	√	√	√	8
Study 22	√	√	√		√	√	√	√	7
Study 23	√	√	√	√	√	√	√	√	8
Study 24	√	√	√	√	√	√	√	√	8

### 2.5 Data synthesis

A comprehensive narrative synthesis was conducted in the data synthesis process for this systematic review on the long‐term effects of childhood experiences on older individuals' health (Table 2). This synthesis involved summarizing and integrating the findings from all the included studies. The data synthesis began by systematically examining the characteristics of the included studies. By adopting a thematic synthesis approach, common themes and patterns across the studies were identified and analyzed. These themes centered around various childhood experiences and their impact on the well‐being of older individuals. A narrative format effectively summarized and presented these themes, providing a holistic overview of the evidence. Furthermore, the quality and risk of bias of the included studies were rigorously assessed to ensure the reliability of the findings. This evaluation allowed for a comprehensive understanding of the strengths and limitations of the available evidence.

**TABLE 2:** Characteristics of included studies.

Study ID	Author, year	Country	Participant age	Study design	Objectives	Findings
Study 1	Asyraf et al. (2021)	Malaysia	Older adults aged 60 years and above	Cross sectional study	To examine the association between adverse childhood experiences (ACEs) and elder abuse among older adults aged 60 years and over in Malaysia	Effect of childhood circumstances on cognitive abilities in older adults
Study 2	Kim et al. (2021)	United States	Aged 18 years and older aged (*N* = 36,309); and 55 or older age groups (*N* = 11,386)	Cross sectional study	To investigates distinct patterns of ACEs in a representative sample of US older adults, and how the ACEs patterns relate to major depression and substance use disorder (SUD)	Relationship between childhood circumstances and mental health in older adults
Study 3	Kwak and Ahn (2020)	Korea	Older adults aged 60 years and more (*N* = 4472)	Korean welfare panel study	To understand the association of childhood adversity with suicidal ideation in later life, and whether gender differences in the effects of childhood adversity exist	Association between ACEs and health issues
Study 4	Xiang and Wang (2021)	United States	Aged 51 years and older (*N* = 16,946)	Longitudinal panel study	To examine the relationship between childhood adversities and major depression in older adults	Connection between childhood trauma and psychiatric disorders in later life
Study 5	Yang et al. (2020)	China	Aged 45 years and older	Longitudinal study	To investigate various aspects of childhood adversity influencing depressive symptoms later in life	Role of childhood abuse and neglect in shaping adult mental health
Study 6	Hu and Wei (2022)	China	Aged 60 and over with long‐term care needs (*N* = 2186)	China health and retirement survey	To investigate the relationships between childhood adversities and unmet long‐term care needs of older adults in China and the mediation effects of family relationships	Role of chronic stress in the development of age‐related illnesses
Study 7	Lin et al. (2021)	China	Aged 45 years or older (*N* = 11,972)	Cross sectional study	To examine associations between ACEs and subsequent chronic diseases and to assess whether age, sex, educational level, annual per capita household expenditure level, and childhood economic hardship modify these associations	Impact of childhood nutrition and health on adult health outcomes
Study 8	Alkhalidy et al. (2021)	Jordan	18 years or above (*N* = 4195)	Cross sectional study	To use obesity measures, body mass index and waist circumference (WC) to predict the CVD and T2D risk and to determine the best predictor of these diseases among Jordanian adults	Impact of childhood nutrition and health on adult health outcomes
Study 9	Sasaki and Carpenter (2022)	United States	Aged 60 years and older (*N* = 3042)	National health and nutrition examination survey (NHANES)	To understand which metals or metabolites have associations with cognitive function tests relating to immediate learning and recall, delayed recall, and working memory in older adults using data from the NHANES	Influence of early exposure to environmental toxins on long‐term health
Study 10	Larkin et al. (2017)	United States	Older adults aged 55 and older	Cross sectional study	To examine the relationship between ACEs and substanceuse among older adults living in public housing	Influence of early exposure to environmental toxins on long‐term health
Study 11	Merrick et al. (2017)	United States	Aged 18 or older (*N* = 7465)	Cross sectional study	To investigate the relationship between an expanded adverse childhood experience (ACE) score that includes being spanked as a child and adult mental health outcomes by examining each ACE separately to determine the contribution of each ACE	Association between ACEs and health issues
Study 12	Sonu et al. (2019)	United States	Aged ≥4, 18–34, 35–54 and ≥55 years	Cross‐sectional analysis	To examine the association of ACEs with early‐onset chronic conditions	Association between ACEs and health issues
Study 13	Knaevelsrud et al. (2017)	Germany	Aged between 63 and 85 years	Randomized Controlled Trial	To investigate the efficacy and feasibility of an Internet‐based, therapist‐guided cognitive‐behavioral therapy (Internet‐based CBT) for older individuals with posttraumatic stress symptoms	Influence of early‐life adversity on mental health outcomes
Study 14	Friedman et al. (2017)	United States	Aged 60 and older	Retrospective analysis	To describe victim characteristics and determinants of recurrent physical abuse of elderly	Connection between childhood trauma and psychiatric disorders in later life
Study 15	Copeland et al. (2018)	United States	Aged 9–16, 19, 21, 25 and 30 years	Population‐based cohort study	To assess the association between cumulative childhood trauma exposure and adult psychiatric and functional outcomes	Connection between childhood trauma and psychiatric disorders in later life
Study 16	Llabre et al. (2017)	United States	Aged 18 to 74	Community health study	To report the prevalence of ACEs in Hispanics/Latinos in the US and their association with major risk factors and diseases in adulthood	Connection between childhood trauma and psychiatric disorders in later life
Study 17	Zhang et al. (2018)	China	Aged 45 years and older	Health and retirement study	To examined the association between childhood conditions and cognitive function among middle‐aged and older adults in China	Long‐term consequences of childhood poverty and social disadvantage
Study 18	Dannehl et al. (2017)	Germany	N/A	Longitudinal study	To examine whether different kinds of childhood adversity might account for cognitive deficits in patients with major depression	Cognitive development and early‐life experiences
Study 19	Lyall et al. (2018)	United kingdom	Aged 37–73 years	Cross sectional study	To examine the association between objectively assessed circadian rhythmicity and mental health and wellbeing phenotypes, including lifetime history of mood disorder	Impact of education and intellectual stimulation during childhood
Study 20	Saleh et al. (2017)	United States	Aged 20–50 years	Cross sectional study	To investigate the effects of early life stress on depression, cognitive performance and brain morphology	Impact of education and intellectual stimulation during childhood
Study 21	Domènech‐Abella et al. (2017)	Spain	Aged 18–49, 50–79, and 80+ years (*N* = 4753)	Cross sectional study	To investigate the influence of social networks in the relationship between loneliness and depression in the older adult population in Spain	Impact of early attachment and familial relationships on adult social support
Study 22	Choi et al. (2017)	United States	*N* = 14,738 for the 50+ age group	Cross sectional study	To examine the association between ten types of ACEs and lifetime mental and substance use disorders (MSUDs) among those aged 50+	Impact of early attachment and familial relationships on adult social support
Study 23	Taylor et al. (2018)	United States	Aged 55 years and above (*N* = 1439)	Cross sectional study	To investigate the impact of objective and subjective social isolation from extended family members and friends on depressive symptoms and psychological distress among a national sample of older adults	Relationship between childhood adversity and social engagement in older adults
Study 24	Robb et al. (2020)	United kingdom	Aged 50 years and over	Cross sectional study	To investigate the impact of COVID‐19 and associated social isolation on mental and physical wellbeing among older people	Relationship between childhood adversity and social engagement in older adults

## 3 RESULTS

### 3.1 Physical health

Adverse health effects in older people may have origins in childhood starvation. Stunted growth and decreased development are expected in malnourished children, making them more vulnerable to chronic conditions in their later years.^18^ Malnutrition in childhood is associated with an increased risk of cardiovascular disease, hypertension, type 2 diabetes, and obesity in later life.^4^ Physiological alterations that predispose individuals to various chronic diseases might be triggered by malnutrition during crucial stages of growth and development. Malnutrition also lowers resistance to disease and illness by weakening the body's immune system.^18^

There may be severe consequences for older individuals' health if they are exposed to environmental pollutants from childhood. Due to the ongoing maturation of their organs and systems during infancy and early childhood, young individuals are at a greater risk of experiencing the harmful impacts of environmental contaminants. Childhood exposure to chemicals, including lead, mercury, pesticides, and air pollution, has been related to various health problems in old age.^19^ These toxins may disrupt normal physiological processes and organ function, which have been related to respiratory diseases, neurological disorders, cardiovascular problems, and even cancer. Protecting children and reducing their exposure is paramount because of the potential for environmental toxins to have lifelong consequences.^20^

Furthermore, abuse, neglect, household dysfunction, or witnessing violence are all examples of adverse childhood experiences (ACEs) that can negatively impact physical health during older age.^21^ ACEs have been associated with various health issues, according to several studies.^18^, ^22^ Heart disease, obesity, diabetes, chronic pain, and autoimmune illnesses are only some chronic conditions more likely to strike those who have encountered ACEs.^18^ Exposure to toxic stress in childhood has been linked to long‐term physiological dysregulation and inflammation by disrupting the normal functioning of the body's stress response system.^23^ These chronic stresses increase the likelihood of developing health issues in older individuals.

Moreover, several degenerative diseases associated with old age have been linked to stress, especially early‐life trauma (infancy to late adolescence). Hormones such as cortisol, released in response to stress, can have detrimental effects when present in elevated concentrations during critical developmental stages.^18^ Chronic stress is associated with accelerated aging, immune system dysfunction, and heightened inflammation, particularly during the formative years.^24^ Additionally, poor sleep, unhealthy eating habits, and reduced physical activity are lifestyle factors significantly impacted by chronic stress in individuals from childhood through their later years.^19^ As a result, the body's natural repair and regeneration processes are hampered, increasing the likelihood of developing chronic diseases as they progress through life.^24^

### 3.2 Mental health

The impacts of childhood trauma, neglect, or unstable family environments on later‐life mental health have been studied extensively.^18^ Numerous studies have discovered a correlation between ACEs and later‐life psychological distress. Mental health problems such as depression, anxiety disorders, PTSD, and substance abuse can all have their roots in traumatic events that occurred in childhood.^21^, ^22^ Brain development, emotional regulation, borderline personality disorder, and higher susceptibility to mental health challenges are ways early‐life stress and trauma can manifest in an older person's mental and emotional well‐being.^25^

Mental health can be compromised when children are exposed to early adversity. Children from low‐income households are disproportionately affected by environmental risks, which encompass poverty, social isolation, a lack of suitable role models, and high stress levels. These detrimental effects of poverty on a child's brain development, academic performance, and social connections can worsen mental health issues.^26^ Furthermore, neglect and abuse during childhood significantly impact mental health in later life. Trauma resulting from abuse and neglect can impede the development of secure attachment, diminish trust and self‐esteem, and worsen difficulties with emotional regulation.^25^ Any form of abuse, whether physical, sexual, or emotional, inflicted on a child dramatically increases the risk of subsequent psychiatric disorders in older individuals.^27^ Some of the brain regions most susceptible to long‐term alterations following trauma include those involved in emotion regulation, memory, and stress response.^28^ Conditions including severe depression, mania, borderline personality disorder, and dissociative identity disorder may all be exacerbated by these effects.^29^, ^30^ This list of concerning factors underscores the need for trauma‐informed therapies and support for older individuals.^31^

### 3.3 Cognitive function

The circumstances of childhood have a significant impact on the cognitive abilities of older individuals. Early life experiences, such as exposure to enriching environments, cognitive stimulation, and supportive relationships, influence cognitive development. Later in life, children with access to stimulating activities, educational opportunities, and positive social interactions are more likely to have superior cognitive abilities.^32^ However, neglect, maltreatment, or persistent stress throughout childhood have been linked to impaired cognitive development and age‐related memory loss in older individuals.^33^

Education and intellectual stimulation during development substantially affect cognitive function in later life. Literacy, numeracy, and problem‐solving skills are the building blocks of quality education and, by extension, the foundation for a lifetime of learning.^32^ Increased schooling is linked to better cognitive function and lessens the likelihood of cognitive decline with aging.^34^ Intellectual stimulation through activities such as reading, puzzles, and mentally challenging tasks also contributes to maintaining cognitive abilities in old age.^35^ Moreover, a higher risk of cognitive impairment and decline in older persons has been linked to childhood trauma, such as physical or sexual abuse, neglect, or exposure to violence.^27^ Trauma's neurobiological impacts might hinder cognitive processes like memory, attention, and executive function. The chance of developing Alzheimer's disease, dementia, and mild cognitive impairment increases among people who have experienced trauma.^28^

Socioeconomic factors significantly influence cognitive aging. A child's SES, including factors such as parental income, education, and occupation, can influence cognitive capacities in older individuals.^36^ Children from disadvantaged socioeconomic circumstances frequently have limited access to quality education, health care, and cognitive stimulation, contributing to cognitive disadvantages that persist into old age.^30^ In addition, socioeconomic factors can influence other cognitive health determinants, such as nutrition, stress levels, and access to health care, thereby further influencing cognitive aging outcomes.^32^

### 3.4 Social relationships

Childhood experiences, particularly early attachment experiences and familial relationships, play a crucial role in forming social connections in later life. Secure attachments with caregivers during childhood provide a foundation for healthy interpersonal relationships later in life.^37^ Individuals who have experienced positive and secure attachments are more likely to develop trusting, supportive, and satisfying relationships with peers, friends, and romantic partners.^38^ These secure relationships provide a solid social support network linked to better overall well‐being and resilience in facing elder life's challenges.

Childhood social isolation or neglect can have enduring effects on social connections in older individuals. Children who experience social isolation or lack social support may struggle to form and maintain relationships later in life.^39^ They may struggle to develop social skills, build trust, and engage in healthy social interactions. The absence of positive social connections during childhood can lead to social withdrawal, loneliness, and limited social networks in older individuals, negatively impacting mental health and overall quality of life.^40^

Childhood trauma, such as abuse, neglect, or exposure to violence, can significantly impact social relationship quality in older individuals.^38^ Individuals who have experienced childhood trauma may face challenges forming and maintaining healthy, trusting, and intimate relationships.^26^ They may have difficulties with emotional regulation, trust, and communication, which can affect the depth and quality of their social connections. Childhood trauma can also contribute to relationship difficulties, such as fear of intimacy, emotional detachment, or patterns of unhealthy relationship dynamics.^25^

Childhood SES influences the formation and composition of social networks among older individuals. Children from higher SES backgrounds often have greater access to resources, educational opportunities, and social networks, which can enhance their social engagement and network size.^4^ In contrast, children from lower SES backgrounds may face social disadvantages, limited resources, and reduced access to social support.^37^ These socioeconomic disparities can persist in older individuals, impacting social network size, quality, and the ability to access supportive relationships.^24^

## 4 DISCUSSION

This systematic review delves into the far‐reaching consequences of ACEs on the lives of older individuals, addressing the domains of physical health, mental health, cognitive function, and social relationships. The findings of this review demonstrate that ACEs have a profound and enduring impact on older individuals across various aspects of their lives.

Firstly, this review uncovers a substantial relationship in the realm of physical health, indicating a strong association between ACEs and a wide range of chronic diseases later in life. It is well‐documented that childhood malnutrition can have profound and lasting consequences on physical health. The review's findings are in alignment with previous research, which has consistently shown that malnourished children face a higher risk of developing cardiovascular disease, hypertension, type 2 diabetes, and obesity as they age. Malnutrition during critical developmental stages can lead to physiological changes that predispose individuals to chronic diseases and weaken the immune system, making them more susceptible to illness.^41^, ^42^ This correlation between childhood malnutrition and poor health in later life has been consistent across numerous studies.^4^, ^18^ Moreover, a study by Mwene‐Batu et al.^43^ revealed a significant association between childhood malnutrition and an increased risk of mental health disorders in older age, including depression and anxiety. This underscores the multifaceted impact of childhood malnutrition on overall health outcomes throughout the lifespan.

Furthermore, the review highlighted the potential long‐term effects of childhood exposure to environmental pollutants on older individuals. Children are more vulnerable to the adverse effects of environmental contaminants due to their developing organs and systems. The findings support previous research that has linked childhood exposure to chemicals such as lead, mercury, pesticides, and air pollution to a range of health problems in old age, including respiratory diseases, neurological disorders, cardiovascular issues, and cancer.^44^ It is crucial to emphasize the need for protective measures to reduce childhood exposure to environmental toxins, as the review rightly points out. These findings echo the concerns of environmental health researchers who stress the importance of safeguarding children from pollutants that can have lifelong health consequences.^19^, ^20^

The impact of ACEs on physical health in older age is a subject of considerable interest. This review underlines the association between ACEs and various health issues, including heart disease, obesity, diabetes, chronic pain, and autoimmune illnesses.^18^ These findings align with the broader body of research that has consistently linked ACEs to a higher risk of developing chronic health conditions.^45^ The review also highlighted the role of toxic stress in childhood in disrupting the body's stress response system, leading to long‐term physiological dysregulation and inflammation, which, in turn, contributes to increased health risks in older individuals. A study by Bhutta et al.^46^ demonstrated how toxic stress in childhood can lead to persistent inflammation and immune dysregulation, predisposing individuals to chronic diseases later in life. These mechanisms have been well‐documented in several studies, supporting the conclusions drawn in this systematic review.^18^, ^21^, ^22^, ^23^

Moving on to the realm of mental health, the review emphasizes the profound and enduring impact of childhood trauma, neglect, and unstable family environments on older individuals. The findings align with the results of numerous previous studies that have consistently demonstrated a strong correlation between ACEs and later‐life psychological distress.^47^, ^48^ The review identifies a range of mental health problems, including depression, anxiety disorders, PTSD, and substance abuse, as potential outcomes of traumatic childhood events.^22^ These findings align with the well‐established connection between early‐life stress and the development of mental health challenges in older age. Moreover, the review highlights the potential effects of abuse, whether physical, sexual, or emotional, on the increased risk of psychiatric disorders in older individuals. The review is consistent with the existing literature, which has repeatedly demonstrated the long‐lasting psychological consequences of childhood abuse.^18^, ^21^, ^22^, ^23^, ^24^, ^25^, ^26^, ^27^, ^28^, ^29^

The review also draws attention to the impact of socioeconomic factors on cognitive function in older age. It highlights the crucial role of early‐life experiences, such as exposure to enriching environments and supportive relationships, in influencing cognitive development.^33^ The findings underscore the importance of access to stimulating activities and educational opportunities during childhood in promoting superior cognitive abilities in later life. In contrast, the review also points out that childhood trauma, neglect, or exposure to violence can impair cognitive development and contribute to age‐related memory loss. These findings are in line with previous research that has explored the long‐term cognitive consequences of childhood adversity, supporting the conclusions of this systematic review.^32^, ^33^, ^34^, ^35^, ^49^ The influence of childhood SES on cognitive aging is a critical aspect of the review's findings. The review emphasizes the impact of SES on cognitive capacities in older individuals, highlighting the disparities in access to education, health care, and cognitive stimulation.^27^, ^28^, ^32^ These disparities in childhood can have lasting effects on cognitive abilities in later life. The review also points out that socioeconomic factors can affect other determinants of cognitive health, such as nutrition, stress levels, and access to health care, further influencing cognitive aging outcomes.^33^, ^34^ These findings align with a substantial body of research on the relationship between SES and cognitive aging, underlining the importance of socioeconomic factors in influencing cognitive health in later life.^30^, ^36^, ^50^

In the realm of social relationships, the review underscores the significant role of childhood experiences, including early attachment experiences and familial relationships, in forming social connections in later life. The findings emphasize the positive impact of secure attachments during childhood on the development of healthy interpersonal relationships in older age. These secure relationships provide a robust social support network associated with better overall well‐being and resilience in facing the challenges of old age.^37^, ^38^ The review also highlights the negative effects of childhood social isolation or neglect on social connections in older individuals. The findings suggest that individuals who experience social isolation or lack social support during childhood may struggle to form and maintain relationships later in life.^4^, ^24^ These individuals may face challenges in developing social skills, trust, and healthy social interactions, which can lead to social withdrawal, loneliness, and limited social networks in older age. These findings align with previous studies that have explored the long‐term social consequences of childhood adversity, supporting the review's findings.^39^, ^40^, ^51^, ^52^

## 5 IMPLICATIONS FOR PUBLIC HEALTH AND POLICY

Childhood experiences significantly affect health trajectories and outcomes in older individuals. Understanding this connection has significant public health implications and demands evidence‐based policies and intervention strategies.

### 5.1 Public health implications

The health impacts of childhood experiences extend far beyond individuals, reverberating through communities and societies. Childhood conditions influence older individuals' chronic disease risk, mental health, and even life expectancy, which are crucial public health indicators. Addressing ACEs thus becomes a matter of public health, contributing to population health improvement and reducing health disparities.

### 5.2 Evidence‐based policies

Given the far‐reaching health implications of childhood experiences, policies addressing early‐life conditions are of utmost importance. These should be evidence‐based, meaning they are grounded on and supported by solid scientific data. The goals of these policies can range from alleviating the stresses of poverty to expanding access to high‐quality education and healthcare. Trauma‐informed care and family mental health assistance are two approaches that can be incorporated into these policies to help lessen the impact of ACEs. Addressing the root causes of health disparities linked to childhood experiences is key to promoting health equity and well‐being throughout the lifespan.

### 5.3 Potential intervention strategies

A variety of intervention strategies can be implemented to improve childhood experiences and, by extension, older individuals' health. Poverty reduction is one crucial strategy. Policies that provide financial stability for families—such as livable wage laws, affordable housing initiatives, and social welfare programs—can improve children's living conditions and reduce stressors that negatively impact health.^53^ Provision of trauma‐informed care is another important intervention. This approach involves recognizing and responding to the effects of all types of traumas. In the context of childhood experiences, trauma‐informed care can support children who have experienced adverse events, helping to prevent or mitigate the long‐term effects of this trauma. Lastly, promoting healthy eating habits in childhood can contribute to better older individuals' health. Policies that increase the availability of good, inexpensive food in all communities and public health initiatives aimed at children and their families can help achieve this goal.

## 6 RECOMMENDATIONS

Based on this systematic review of the long‐term effects of childhood experiences on older individuals, several critical areas for further research become apparent. First and foremost, there is a compelling need for more longitudinal studies that follow individuals from childhood into older age. These longitudinal studies offer a dynamic perspective on how childhood experiences cumulatively impact health over time and can reveal the intricacies of this relationship. Diversity in research is pivotal. Future studies should strive to encompass diverse populations, considering variations in cultural, socioeconomic, and geographic contexts. Understanding how the impact of childhood experiences differs among these diverse groups can pave the way for more tailored interventions.

To deepen our understanding, research should delve into the underlying mechanisms of influence. Investigating the biological, psychological, and social pathways through which childhood experiences affect health in older age can yield critical insights for the development of targeted interventions. Exploring intergenerational effects is equally imperative. Research should center on how the experiences of one generation influence the health of the next, and how interventions can break the cycle of adversity, thereby interrupting the chain of adverse outcomes.

## 7 CONCLUSION

The effects of adversity in childhood persist throughout older people's life. Adversity in childhood, such as abuse, neglect, or socioeconomic deprivation, is linked to an increased risk of developing chronic diseases and mental health disorders in later life. On the other hand, healthy older people can attribute their happier and healthier childhoods to parental care, academic achievements, and secure attachments. Recognizing the influence of early life experiences on the health of older individuals highlights the significance of programs and policies that prioritize the well‐being of children. Investing in children's education and fostering nurturing environments can contribute to the long‐term health of individuals and communities.

## AUTHOR CONTRIBUTIONS

Data were extracted by MRP, MKKR, and FTJ. Any disagreements were resolved by MRP, MKKR, FTJ, FA, MW, KA, MD, SM, MDe, and MU. Moreover, MRP, MKKR, FA, MU, MD, SM, and MDe conducted critical analysis. MRP, MKKR, KA, and MW prepared the manuscript draft. All authors contributed to the in‐depth revisions of the manuscript and approved the final version.

## FUNDING INFORMATION

There was no external fund taken for this current research.

## CONFLICT OF INTEREST STATEMENT

The authors have no competing interest at all.

## Data Availability

Data sharing is not applicable to this article as no new data was created or analyzed in this study.
